# Chemotherapy- and Irradiation-Induced Bone Loss in Adults with Solid Tumors

**DOI:** 10.1007/s11914-015-0266-z

**Published:** 2015-02-25

**Authors:** Michel D. Wissing

**Affiliations:** Department of Medical Oncology, Leiden University Medical Center, Albinusdreef 2, Leiden, 2333 ZA The Netherlands

**Keywords:** Chemotherapy-induced bone loss, Radiotherapy-induced bone loss, Solid tumors, Antiresorptive agents, Radionuclides

## Abstract

It is estimated that bone loss occurs in 70 % of all patients dying from cancer, causing a significant disease burden in cancer patients. Bone loss is caused by cancer itself and its metastases, but also by cancer therapies. Of the cancer therapy-induced bone loss, hormone therapies are best known for their bone damaging abilities. However, chemo- and radiotherapy may result in bone loss too. In this review, direct and indirect effects of various chemotherapies (such as methotrexate, imatinib, and taxanes) that cause bone loss are discussed. Furthermore, we discuss bone loss caused by radiotherapy and radionuclides, of which the latter may be reduced with the introduction of the alpha-emitter Radium-223. Finally, agents preventing chemotherapy- or radiotherapy-induced bone loss, in particular denosumab and bisphosphonates, are being reviewed for their efficacy in preventing chemotherapy- and irradiation-induced bone loss in cancer patients.

## Introduction

Cancer is one of the most prevalent and deadliest diseases in the world, with an estimated 1.7 million new cases and 586,000 deaths in the USA in 2014 [1]. In cancer patients, bone loss occurs frequently: it is estimated that bones are affected of over 70 % of all patients dying from cancer, often resulting in significant morbidity and mortality [2]. Bone disease primarily occurs due to bone metastases: lung carcinomas, causing most cancer deaths in both men and women [1], as well as prostate and breast cancer, the most prevalent cancers in men and women, respectively [3], frequently metastasize to the bones; other solid tumors metastasize to bones as well [2]. Furthermore, bone may be damaged in cancer patients by other causes, such as cancer therapy. For example, in a case–control study, breast cancer patients without bone metastases had a significant increase in vertebral fractures (odds ratio (OR) of 4.7) as compared to controls from a general population [4].

It is well known that hormonal suppression by hormone ablation therapy, frequently used in patients with among others prostate and breast cancer, results in osteoporosis and bone fractures due to a decrease in bone mineral density [5]. In prostate cancer patients who received long-term androgen-deprivation therapy, osteoporosis rates increased from 35.4 % in hormone-naive patients to 80.6 % of patients treated with androgen-deprivation therapy for ten or more years [6]. In a study with 50,613 prostate cancer patients who did and did not receive androgen-deprivation therapy, androgen-deprivation therapy increased the risk of fractures from 12.6 to 19.4 % [7]. Similarly, hormonal therapy in breast cancer patients, particularly treatment with aromatase inhibitors such as letrozole and exemestane, has been found to increase the risk for osteoporosis and (pathological) fractures [8, 9]. Considering the role of hormones in bone physiology, aforementioned increased occurrences of bone loss and skeletal-related events after hormonal-ablation therapy is not surprising.

Treatment of cancer patients with other therapies, such as radio- and chemotherapy, may result in significant bone loss as well. These therapies may directly target the bones or may provoke bone loss by indirect systemic effects. Furthermore, agents currently administered to cancer patients aiming to reducing bone-related adverse events may actually result in osteonecrosis. In this review, the prevalence and (potential) mechanisms of bone loss after administration of chemotherapy and irradiation will be discussed. Furthermore, novel modalities that may reduce chemotherapy- or irradiation-induced bone loss will be reviewed.

## Chemotherapy and Bone Loss

Chemotherapy may lead to bone damage via indirect systemic effects, of which the most studied effect is the loss of ovarian function in women. In one study, adjuvant chemotherapy with cyclophosphamide, methotrexate, and fluorouracil in premenopausal women with breast cancer resulted in chemotherapy-induced amenorrhea in 68 % (95 % CI 66–70 %) of these patients [10]. This ovarian failure resulted in rapid bone loss: within 2 years, this combination of chemotherapy resulted in bone loss of 9.5 % in the lumbar spine and 4.6 % in the femoral neck [11]. Other combinations of adjuvant chemotherapy induce amenorrhea in premenopausal breast cancer patients as well [12, 13•].

However, chemotherapy may also have a direct impact on bone (re)modeling. As summarized by Hadji et al., studies evaluating adjuvant chemotherapy in premenopausal breast cancer patients consistently reported a decrease in bone mineral density during the first year after initiation of therapy [13•]. For example, one study with premenopausal breast cancer patients reported that bone mineral density in the spine and hips of women during 6 months’ adjuvant systemic chemotherapy was decreased by 1.01–1.05 g/m^2^, independently of changes to ovarian function or amenorrhea [14].

Imatinib, used for the treatment of gastrointestinal stromal tumors and leukemia, directly targets various receptors that play a role in the bone microenvironment, such as the platelet-derived growth factor (PDGF) receptor and the macrophage colony stimulating factor (c-Fms) receptor [15, 16]. In manipulating these receptors, bone formation was found to be increased by increasing osteoblast activity at metaphyseal osteochondral junctions and by eliminating osteoclasts from these junctions, leading to decreased bone resorption at the growth plate [17]. On the other hand, imatinib increased osteoclast activity at distal trabecular bone, resulting in increased bone resorption [17].

Many chemotherapies such as taxanes cause myelosuppression [18, 19]. Recently, Quach et al. reported that myelosuppression resulted in bone loss in mice by increased bone resorption, which was associated with increased expression of monocyte chemoattractant protein 1 (MCP1) and other inflammatory cytokines [20•]. MCP1 was also found to be increasingly expressed in cancer patients who had recently received chemotherapy and had bone loss. Inhibition of osteoclast activity by zoledronic acid prevented this MCP1-associated bone loss [20•].

Methotrexate, used for the treatment of, among others, breast cancer, lung cancer, head and neck cancer, choriocarcinoma, and osteosarcoma, directly targets bone tissue too. In an in vivo experiment, the anti-metabolite increased apoptosis of osteocytes by a 4.3-fold, while increasing the number of osteoclasts by a 1.8-fold, associated with increased expression of the inflammatory cytokines IL-6 and IL-11 [21]. These changes resulted in a 35 % loss of trabecular bone. In a different study, it was found that methotrexate-induced changes to bone (re)modeling may occur due to decreased activation of the Wnt/β-catenin signaling pathway [22].

Finally, corticosteroids such as prednisone play an important role in the treatment of patients with, among others, prostate cancer and multiple myeloma. Although the antitumor activity of this glucocorticoid as a single agent is being debated in most tumors, it is regularly administered concomitantly with other antitumor agents, such as taxanes and abiraterone. In multiple myeloma, the antitumor efficacy of corticosteroids by themselves is evident due to its ability to inhibit IL-6 production and to induce apoptosis in plasma cells [23]. However, prednisone directly affects bone tissue: osteoblast activity is reduced by decreased osteoblast cell replication and differentiation, while apoptosis is increased; osteoclastogenesis is increased by increased expression of RANK-ligand and decreased expression of osteoprotegerin, a decoy receptor of RANK-ligand [24]. Furthermore, urinary excretion of calcium is increased, while intestinal absorption of calcium is inhibited, resulting in further bone damage [23].

## Radiotherapy and Bone Loss

On the contrary to chemotherapy, which causes bone loss both by direct and indirect effects, radiation primarily causes direct damage to bone tissue. In pediatrics, most damage from radiotherapy occurs to the growth plate, causing growth disturbances, while in adults, osteopenia and incomplete healing of bone damage results in skeletal-related events such as insufficiency fractures [25]. The exact pathogenesis of radiotherapy-induced bone loss has not been unraveled completely yet. Consensus exists that radiation decreases the number of active osteoblasts by arresting these in the cell cycle, altering their ability to differentiate, and sensitizing the cells to apoptosis [26, 27, 28•]. As summarized by Chandra et al., the role of radiation therapy in the activation of osteoclasts is still being debated, with some studies reporting a decrease in osteoclast activity while others report an increase in number and activity of osteoclasts [28•]. Nevertheless, the hypothesis that radiotherapy may induce osteoclast activity is strengthened by a clinical study that found that the addition of antiresorptive agents such as zoledronic acid prevented or delayed skeletal-related events in patients with bone metastases, and by in vivo studies that indicated a decrease in radiotherapy-induced bone loss when mice were treated with bisphosphonates [29–31]. A recently published study suggests that PTH1-34 may prevent radiotherapy-induced bone loss by preventing apoptosis of osteoblasts and osteocytes [28•].

## Radionuclides and Bone Loss

Radionuclides, such as Rhenium-186, Strontium-89, and Samarium-153, localize to areas with increased bone turnover, thereby selectively targeting osteoblastic bone metastases [32, 33]. For this reason, radionuclides have been used to treat bone metastases in a variety of cancers, such as prostate and breast cancer. However, most radionuclides are beta-emitters and/or even gamma-emitters, which are moderately to highly penetrating, thereby damaging surrounding tissue as well. Indeed, it has been shown that these radionuclides result in damage of healthy bone marrow, as noted by the increase in thrombocytopenia and leucopenia [33]. Therefore, radionuclides most likely damage healthy bone tissue as well, although this has not been proven. Such damage could be reduced by making use of alpha-emitting particles, which are highly energetic but do not have a high penetrative capacity. Radium-223 chloride is such a particle. It has received approval by the United States Food and Drug Administration (US FDA) for the systemic treatment of patients with castrate-resistant prostate cancer with bone metastases in 2013. As described previously, Radium-223 emits four alpha-particles and two beta-particles during its decay, until it stabilizes as Lead-207, thereby selectively targeting cells in its direct surroundings [34•]. Radium-223 increased overall survival in mCRPC patients while bone marrow toxicity was relatively low as compared to other radionuclides [35]. Nevertheless, these results need to be confirmed in studies assessing long-term efficacy and toxicity of radium-223 treatment. Currently, clinical trials are being performed to study the antitumor efficacy in patients with cancers metastasized to bones other than prostate cancer, and in patients with primary bone cancer.

## Agents Used for the Prevention of Bone Loss

It is generally thought that the key to cancer-induced bone loss is an increase in osteoclast activity, resulting in decreased bone mass. Over the past two decades, bisphosphonates and the RANK ligand inhibitor denosumab have become available to prevent both cancer-induced bone loss and cancer therapy-induced bone loss. Bisphosphonates reduce osteoclast activity, thereby increasing bone mass, resulting in increased strength of the bone and a reduction in pathological fractures [36, 37]. Various bisphosphonates have been approved for bone-related diseases, including ibradronic acid, pamidronic acid, risedronate, and zoledronic acid for the reduction of skeletal-related events in cancer patients and for patients with multiple myeloma. Of these, zoledronic acid is most commonly used, as various studies in patients with cancer-related bone disease indicated superiority of zoledronic acid over other bisphosphonates [38–40]. Treatment with bisphosphonates decreases pain secondary to bone metastases, pathological fractures, and other skeletal-related events, thereby improving quality of life [41–43].

Denosumab is a subcutaneously administered, monoclonal antibody approved by the US FDA for the treatment of unresectable giant cell tumor of bone in adults and skeletally mature adolescents, for cancer patients at high risk for fracture for example due to androgen-deprivation therapy or adjuvant aromatase inhibitor therapy, and for the prevention of skeletal-related events in patients with bone metastases from solid tumors [44]. In various phase III studies with patients with bone metastases from solid tumors, denosumab was more effective in delaying or preventing skeletal-related events and pain progression than bisphosphonates [45–49]. In prostate cancer patients, denosumab also reduced the risk of symptomatic skeletal events, a biomarker considered more accurate for assessing clinical benefit in patients [50•]. Furthermore, in patients with metastatic lung cancer, overall survival was improved when patients were treated with denosumab as compared to zoledronic acid [51]. However, due to its higher cost, the cost-effectiveness of denosumab as compared to bisphosphonates remains unclear, and many physicians continue to treat cancer patients with bone disease with bisphosphonates [52].

Although bisphosphonates and denosumab aim to reduce bone disease, these agents may also cause bone damage, including hypocalcaemia, atypical femur fractures, and osteonecrosis of the jaw [37, 53]. Osteonecrosis of the jaw occurs in an estimated 7 % (range 0–27.5 %) of all patients treated with bisphosphonates; its mean incidence was 1.7 % in recent studies in which patients were treated with denosumab but did not differ significantly from the incidence of osteonecrosis of the jaw after treatment with bisphosphonates. Although this painful and potentially debilitating adverse event may initially be treated with antibiotics, the damage is often irreversible for which surgical management is needed. It is hypothesized that osteonecrosis of the jaw after therapy with antiresorptive agents is caused by oversuppression of osteoclast activity and/or by compromising of angiogenesis, thereby resulting in bone ischemia and sclerosis [54]. Other factors may contribute to osteonecrosis of the jaw, such as infections, poor oral hygiene, surgery to the jaw bones, diabetes mellitus, smoking, dental extraction, and concurrent medications like glucocorticoids or antiangiogenic medication (among others bevacizumab, sunitinib, sorafenib, mTOR inhibitors) [54, 55••]. Indeed, recent studies have indicated that the incidence of osteonecrosis of the jaw during therapy with bisphosphonates or denosumab can be decreased by improving oral hygiene, by eliminating or stabilizing oral disease before initiating treatment, and by temporarily discontinuing treatment after extensive oral surgery [53, 55••].

Other agents have been or are currently being investigated for their use in the prevention of bone loss, with limited success. For example, studies are ongoing to investigate the use of gonadotropin-releasing hormone agonists such as triptorelin for the prevention of chemotherapy-induced ovarian failure. However, a prospective randomized clinical trial in patients with lymphoma did not find a statistically decreased risk of ovarian failure [56]. A meta-analysis of studies performed in breast cancer patients reported a significant decrease in premature ovarian failure after treatment with a gonadotropin-releasing hormone agonist (RR 0.40, 95 % CI 0.21–0.75), but no effect on resumed menses [57]. A recent study confirms this decrease in premature ovarian failure in breast cancer patients treated with adjuvant chemotherapy [58]. However, long-term studies need to be performed to assess whether such therapy results in a decrease in chemotherapy-induced bone disease.

## Conclusions

Bone disease causes high rates of morbidity and mortality in cancer patients. It can be caused both by the tumor itself and by cancer therapy. Both hormonal therapy, chemotherapy, and radiotherapy may induce bone loss. While radiotherapy-induced bone loss is primarily caused by direct bone damage, chemotherapy-induced bone damage may be the result of direct bone targeting or by indirect systemic effects, such as decreased ovarian function. Multiple agents, such as bisphosphonates and denosumab, have become available to reduce bone damage after antitumor therapy. However, these agents may induce severe bone damage too, particularly osteonecrosis of the jaw. Further research is needed to decrease the disease burden from therapy-induced bone loss in cancer patients.
